# Rapid Biotyping Discrimination of Methicillin-Susceptible and -Resistant *Staphylococcus aureus* Using Flow Injection Analysis–Ion Mobility–Mass Spectrometry

**DOI:** 10.21627/vx9rfz41

**Published:** 2026-05-18

**Authors:** Muhammad Ramzan, Michael W. Christopher, Autumn S. Dove, Kwangcheol C. Jeong, Daniel M. Czyz, Syed Ghulam Musharraf, Timothy J. Garrett

**Affiliations:** Department of Pathology, Immunology, and Laboratory Medicine, College of Medicine, University of Florida, Gainesville, FL 32611, United States; HEJ Research Institute of Chemistry, International Center for Chemical and Biological Sciences, University of Karachi, Karachi 75270, Pakistan; Department of Pathology, Immunology, and Laboratory Medicine, College of Medicine, University of Florida, Gainesville, FL 32611, United States; Emerging Pathogens Institute, University of Florida, Gainesville, FL 32611, United States; Department of Biomedical Sciences and Pathobiology, Virginia-Maryland College of Veterinary Medicine, Virginia Tech, Blacksburg, VA 24060, United States; Emerging Pathogens Institute, University of Florida, Gainesville, FL 32611, United States; Department of Animal Sciences, University of Florida, Gainesville, FL 32611, United States; Department of Microbiology and Cell Science, University of Florida, Gainesville, FL 32611-0700, United States; HEJ Research Institute of Chemistry, International Center for Chemical and Biological Sciences, University of Karachi, Karachi 75270, Pakistan; Dr. Panjwani Center for Molecular Medicine and Drug Research, International Center for Chemical and Biological Sciences, University of Karachi, Karachi 75270, Pakistan; Department of Pathology, Immunology, and Laboratory Medicine, College of Medicine, University of Florida, Gainesville, FL 32611, United States

**Keywords:** flow injection analysis mass spectrometry, high-resolution mass spectrometry, methicillin resistance, methicillin-resistant *Staphylococcus aureus*, methicillin susceptibility, methicillin-susceptible *Staphylococcus aureus*

## Abstract

**INTRODUCTION:**

Antimicrobial resistance is a leading contributor to global mortality. Among the most concerning pathogens is *Staphylococcus aureus*, a gram-positive bacterium capable of causing severe infections. Particularly problematic is methicillin-resistant *S. aureus* (MRSA), which exhibits extensive resistance to antibiotics. Rapid differentiation between resistant and susceptible strains is essential for early and accurate intervention, enabling timely administration of appropriate antibiotics. However, conventional diagnostic methods remain slow, labor-intensive, and costly.

**METHODS:**

In this study, we developed a method using flow injection analysis–ion mobility–high-resolution mass spectrometry for the rapid discrimination of MRSA from its susceptible counterpart, methicillin-susceptible *S. aureus* (MSSA). The total instrument run time is 2 min, and samples can be analyzed after 6 h of incubation.

**RESULTS:**

MRSA was distinguished from MSSA based on the detection of three peptide peaks at m/z 733.791, 763.176, and 769.129, proposed as marker peaks for differentiating MRSA from MSSA. These peptide peaks were identified as phenol-soluble modulins (PSMα1, PSMα2, and PSMα4) using HRMS by applying de novo sequencing from HRMS/MS data.

**CONCLUSION:**

Our study may facilitate the rapid detection and discrimination of *S. aureus* strains, potentially enabling timely intervention and therapeutic strategies for affected patients.

## Non-Specialist Summary

Antimicrobial resistance is a major health threat, and methicillin-resistant *Staphylococcus aureus* (MRSA), a hard-to-treat form of *Staphylococcus aureus*, is a key concern. Standard tests to determine whether a strain is drug-resistant are slow and expensive. This study introduces a rapid lab method that, after only 6 h of growth and a 2 min measurement, identifies three peptide markers unique to MRSA. This could allow much faster detection of resistant strains and quicker, more targeted treatment for patients.

## 1. Introduction

Antimicrobial resistance (AMR) has emerged as one of the most critical public health issues [1]. AMR is expected to cause 10 million fatalities annually by 2050, emphasizing the importance of rapid and accurate identification of microorganisms [2].

*Staphylococcus aureus* is an opportunistic pathogen that colonizes about one-third of people worldwide and may result in invasive disorders such as endocarditis, skin and soft tissue infection, bacteremia, pneumonia, osteomyelitis, and medical implant–associated infection [3]. *S. aureus* infections are mostly challenging due to the prevalence of AMR among isolates, the most common and clinically relevant being methicillin-resistant *S. aureus* (MRSA) [4]. MRSA infections are associated with higher mortality and morbidity and longer hospital stays when compared with methicillin-susceptible *S. aureus* (MSSA) infections [5], causing a reported 20,000 deaths in 2018, the most deaths of any antibiotic-resistant bacteria in the United States [6]. Several MSSA lineages can also be extremely virulent, resulting in lethal infections [7, 8].

Therefore, rapid identification of *S. aureus* and discrimination between MSSA and MRSA strains are essential to ensure proper intervention strategies. However, the accurate optimization of treatments requires a comprehensive antibiotic-resistance profile, which may take up to 72 h using current culture-based approaches [9]. Such prolonged diagnostics often lead to empiric therapy, unnecessarily exposing patients to broad-spectrum agents such as vancomycin. The two most common modalities for MRSA testing are culture and polymerase chain reaction (PCR). Rapid sample-to-answer PCR platforms (e.g., GeneXpert) can provide results within about an hour [10], but their high cartridge cost and low throughput limit routine use [11]. Conversely, chromogenic agar culture is cost-effective and widely applied, particularly for the high-volume colonization screening of inpatients and long-term care residents [10], but it is labor-intensive and requires at least 18–24 h of incubation [12]. Consequently, new methodologies with increased efficiency and less labor-intensive screening are desperately needed [2].

Mass spectrometry (MS) is a promising analytical technique for the investigation of microorganisms because of its advantages in sensitivity, specificity, and speed [13]. MS-based bacterial screening has been mostly achieved by utilizing matrix-assisted laser desorption ionization time-of-flight (MALDI-TOF MS) [14]. The commercially available MALDI Biotyper (Bruker Daltonics, Billerica, MA) and VITEK MS (bioMérieux S.A., Craponne, France) systems [15, 16], approved by the US Food and Drug Administration, can identify microbes at the species level but struggle to distinguish antimicrobial-resistant types [17]. As a result, MALDI-TOF MS is followed by the detection of antibiotic susceptibilities using traditional techniques. Such a delay in diagnostics makes it difficult to rapidly select proper antimicrobial therapies [18]. Several ionization approaches use membrane lipids as diagnostic markers for discriminating different bacteria [19, 20]. However, lipids might have an array of isomers due to changes in headgroup, chain length, *sn*-position, the position of the double bond, and configuration, making structural studies difficult [21, 22].

An alternative rapid analytical technique is offered by flow injection analysis mass spectrometry (FIA-MS), in which the samples are injected into the ionization source of an MS without any chromatographic separation. The utilization of FIA-MS with contemporary high-resolution MS (HRMS), such as Orbitrap and TOF, makes it possible to identify hundreds to thousands of m/z features in biological samples [23, 24]. Compared with liquid chromatography (LC) analysis, FIA may be utilized to deliver a sample aliquot to the MS without chromatographic separation, thereby resulting in run times from 20 s to 2 min, significantly reducing the analysis time and solvent used.

Ion mobility spectrometry (IMS) is a gas-phase method that separates and distinguishes structurally distinct compounds based on their drift time and can provide collisional cross sections using collisions between analyte ions and the inert buffer gas under the influence of a weak electric field [25]. Traveling wave ion mobility spectrometry (TWIMS) differs from other IMS techniques by employing an oscillating electric field to generate voltage “waves” along the ion path, propelling ions through inert gas collisions. Structures for lossless ion manipulation (SLIM) technology build upon the oscillating fields used in TWIMS and incorporate additional applied potentials across sandwiched printed circuit boards. This configuration enables a nonlinear (serpentine) ion path, thereby increasing resolution through extended path length and offering high-resolution ion mobility (HR-IM) [26].

Therefore, the current study was conducted to develop a method for rapid discrimination between MSSA and MRSA using a flow injection analysis–ion mobility–high-resolution mass spectrometry (FIA-IM-HRMS) approach.

## 2. Materials and Methods

### 2.1. Chemicals

All chemicals used in the study were 99.99% pure. LC-MS grade ethanol, formic acid (FA), acetonitrile (ACN), and water (H_2_O) were purchased from Fisher Chemical (Fair Lawn, NJ, United States). A low-concentration electrospray ionization (ESI) tuning mix was purchased from Agilent Technologies (Santa Clara, CA). Difco Tryptic soy broth (TSB) was purchased from Becton Dickinson (Franklin Lakes, NJ, United States).

### 2.2. Collection of Bacterial Strains

The wild-type *S. aureus* 12600 (MSSA) was obtained from the American Type Culture Collection (ATCC 12600). The resistant *S. aureus* 131 (MRSA, HM-466), *S. caprae* M23864:W1 (HM-143), *S. lugdunensis* M23590 (HM-141), *S. epidermidis* BCM0060 (HM-140), *S. capitis* SK14 (HM-117), and *S. warneri* SK66 (HM-120) strains were obtained from the Biodefense and Emerging Infection Research Resources Repository. The clinical isolates (n = 20, including 10 MSSA and 10 MRSA) were obtained from the Kwangcheol C. Jeong Lab at the Emerging Pathogens Institute, University of Florida, Gainesville, FL, United States.

### 2.3. Preparation of Inoculum

All bacterial samples were prepared using the same method as previously reported [27]. Tryptic soy agar plates were used to streak for the isolation of bacteria from a glycerol stock and incubated overnight at 37 °C. One colony was inoculated in a culture tube in 5 mL of TSB and incubated overnight at 37 °C, shaking at 220 rpm. The negative sample contained only media, while the blank sample contained ACN with 0.1% FA. The optical density at 600 nanometers was adjusted to 1.0, followed by a dilution to a final bacterial concentration of 1 × 10^8^ cells in 300 µL of TSB. Both wild-type MSSA (ATCC 12600) and MRSA (HM-466) were incubated for different time periods—i.e., 3, 6, and 24 h, while the clinical isolates were incubated for 6 h at 37 °C.

### 2.4. Sample Preparation for IM-MS Analysis

Following the incubation, 900 µL of 70% ethanol and 300 µL of H_2_O were added to the bacteria; the samples were vortexed for 1–2 min, followed by centrifugation at 14,000 rpm for 6 min. The supernatant was discarded, and the pellet was resuspended in 50 µL of 70% FA and 50 µL of ACN; the samples were vortexed for 1–2 min and centrifuged at 14,000 rpm for 6 min, and the supernatant was transferred to high-performance liquid chromatography (HPLC) vials and stored at –80 ℃ for experimental use. This sample preparation approach primarily focuses on the small-molecule portion of the bacterial isolate, which could include the peptidome and other small molecules.

### 2.5. Flow Injection Analysis–Ion Mobility–Mass Spectrometry (FIA-IM-MS) Analysis

Bacterial samples were analyzed using a MOBILion (Chadds Ford, PA) HR-IM system (MOBIE^®^) with quadrupole time-of-flight (QTOF) (Agilent 6545 Series), coupled with an Agilent 1290 system (Agilent Technologies, Santa Clara, CA), with a binary rapid separation pump, column thermostat, and autosampler. Then 10 µL of the sample was flow injected with a flow rate of 0.2 mL/min using an autosampler without any chromatographic separation. A 50:50 mixture of mobile phases consisting of 0.1% FA in H_2_O (A) and 0.1% of FA in ACN (B) was used. Samples were analyzed using positive mode ESI for a total run time of 2 min. Agilent tune mix was used for instrument calibration. QTOF parameters were controlled using Agilent Mass Hunter Data Acquisition software (Agilent Technologies, Santa Clara, CA), while MOBIE^®^ was controlled by EyeOn^®^ software (MOBILion Systems, Inc.). A MOBILion HR-IM data processor (v2.7.7.1) was used for file conversion, while data analysis was performed using an Agilent Mass Hunter IM-MS Browser (v10.0.1).

MS parameters were followed as a mass range between m/z 100–2000. The acquisition rate was set at 8 spectra, the time at 125 ms/spectrum, the capillary voltage at 3500 V, the gas temperature at 225–250 ℃, the flow rate of drying gas (nitrogen) at 3.0–10.0 L/min, and the nebulizer gas pressure at 15–25 psi. Sheath gas temperature was kept at 125–250 ℃ with a flow rate of 3.0–12.0 L/min.

The MOBIE^®^ parameters were set as follows: SLIM top radio frequency (RF) with a frequency of 764 kHz, drive voltage of 290 V, RF^+^ at 283 V_p–p_, and RF^⁻^ at 294 V_p–p_; SLIM bottom RF with a frequency of 799 kHz, drive voltage of 290 V, RF^+^ at 286 V_p–p_, and RF^⁻^ at 297 V_p–p_. The separation traveling wave was set to a frequency of 25 kHz, an amplitude of 50 V_p–p_, a direction of FWD, and a SINE shape. The fill time was 16 ms; the ion trap time, 1.2 ms; the release time, 3.2 ms; and the IMS frame, 1000 ms. Both the filling and release traveling wave frequencies were 15 Hz, with an amplitude of 20 V_p–p_.

### 2.6. Characterization of Marker Compounds

Compound identification was achieved using Orbitrap Exploris 120 (Thermo Scientific, San Jose, CA, United States) coupled to a Vanquish ultrahigh-performance liquid chromatography (UHPLC) system. The instrument calibration was achieved using Pierce^TM^ Flex Mix^TM^ Calibration Solution (Thermo Scientific). A 10 µL volume of the sample was directly injected with the mobile phase (50:50) at a flow rate of 0.350 mL/min. Data-dependent acquisition mode (top five) was used for sample analysis with a total run time of 2.5 min. Thermo Scientific Xcalibur (v4.6.67.17) was used to control instrument parameters, and Qual Browser was used for data analysis. The instrument parameters were as follows: full scan resolution: 30,000 at m/z 200; scan range (m/z): 100–1500; ddMS^2^: tandem mass spectrometry (MS/MS) resolution 15,000 at m/z 200, 30 normalized collision energy (NCE); spray voltage: 3500 V; sheath gas (arbitrary units): 55; temperature of ion transfer tube and vaporizer: 325 ℃ and 350 ℃, respectively.

### 2.7. Method Validation

The method was validated using various species from the *Staphylococcus* genus, including *S. caprae*, *S. lugdunensis*, *S. epidermidis*, *S. capitis*, *S. warneri*, and 20 clinical isolates of *S. aureus*. Ten isolates of each MSSA and MRSA were analyzed in duplicate.

### 2.8. Multivariate Statistical Analysis

Peak lists were created by summing across the entire 2 min collection period and exporting the resulting spectrum for each sample. The data were aligned across samples with an m/z tolerance of 0.025. Multivariate statistical analysis was performed using MetaboAnalyst 6.0. In the software, data were filtered to remove features not present in greater than or equal to 80% of the samples. Any missing values remaining were replaced with one-fifth of the minimum value across samples. The data were further processed using the interquartile range filter, sum normalization, log transformation, and autoscaling. The number of significant features was obtained using Student’s t-test (*p* ≤ 0.05). Then, sparse partial least squares discriminant analysis (sPLS-DA), the variable importance in projection (VIP) score, and heatmap analyses were performed to identify potential biomarkers.

## 3. Results

### 3.1. FIA-IM-HRMS Analysis of *S. aureus* Samples

In our initial analysis of the data, we focused on visual inspection of spectra to identify differences between MRSA and MSSA colonies. The FIA-IM-HRMS analysis of MSSA wild type (*S. aureus* 12600) and MRSA (*S. aureus* 131) revealed spectral differences based on two peaks by comparing the spectra with blank and negative control (growth media) samples after incubation for 24 h. The samples were analyzed in duplicate. A prominent, singly charged peak at m/z 691.613 was found to be present in both MSSA (*S. aureus* 12600) and MRSA (*S. aureus* 131), while a triply charged peak at m/z 733.792 was found only in MRSA samples, as shown in **Figure 1**.

**Figure 1.**
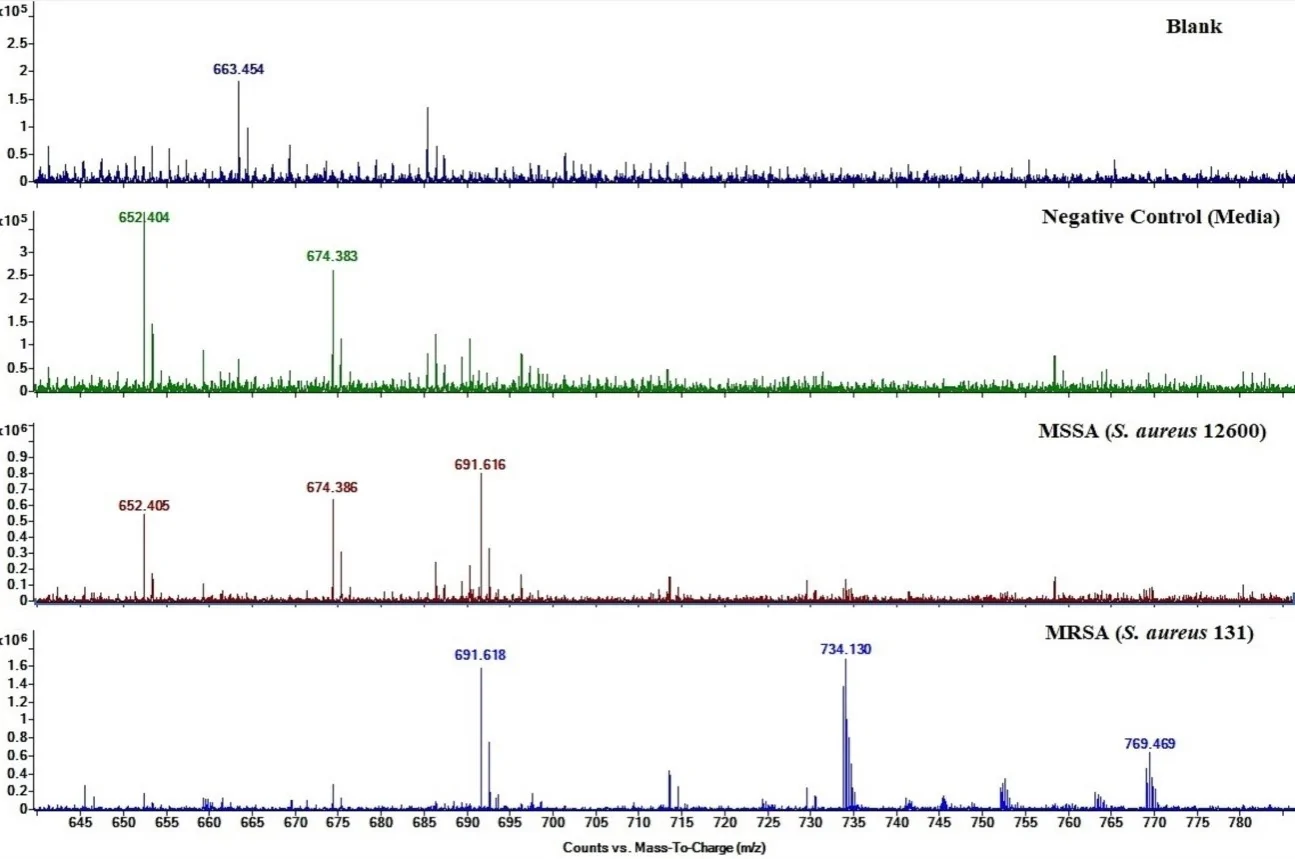
Comparison between the FIA-IM-MS spectra of blank, media, MSSA, and MRSA after 24 h incubation.

### 3.2. Optimization of Assay Time

Visual inspection of the spectra from the FIA-IM-HRMS analysis of MSSA (*S. aureus* 12600) and MRSA (*S. aureus* 131) samples showed that differentiation can be observed based on the peak at m/z 733.792, which was elevated only in MRSA. The next assignment was to optimize an incubation period that could be used for rapid analysis and determine whether the observed peak was dependent on growth time. Therefore, the strains (*S. aureus* 12600 and *S. aureus* 131) were incubated at three different periods (3 h, 6 h, and 24 h) to observe any changes in signal intensity for m/z 733.792 with respect to incubation time. The intensity of the peak was found to increase with increasing incubation time, as represented in **Figure 2**. It can be observed that the peak at 6 h had a higher intensity compared to 3 h and lower than at 24 h. A considerable intensity of the peak was present at the 6 h period, and incubating for 24 h did not substantially increase the peak intensity. We also compared MRSA and MSSA at 6 h and observed that the peak at m/z 733.790 was very low in abundance in the MSSA strain (**Supplemental Figure S1**). Based on these observations, the incubation time at 6 h was selected for further validation.

**Figure 2.**
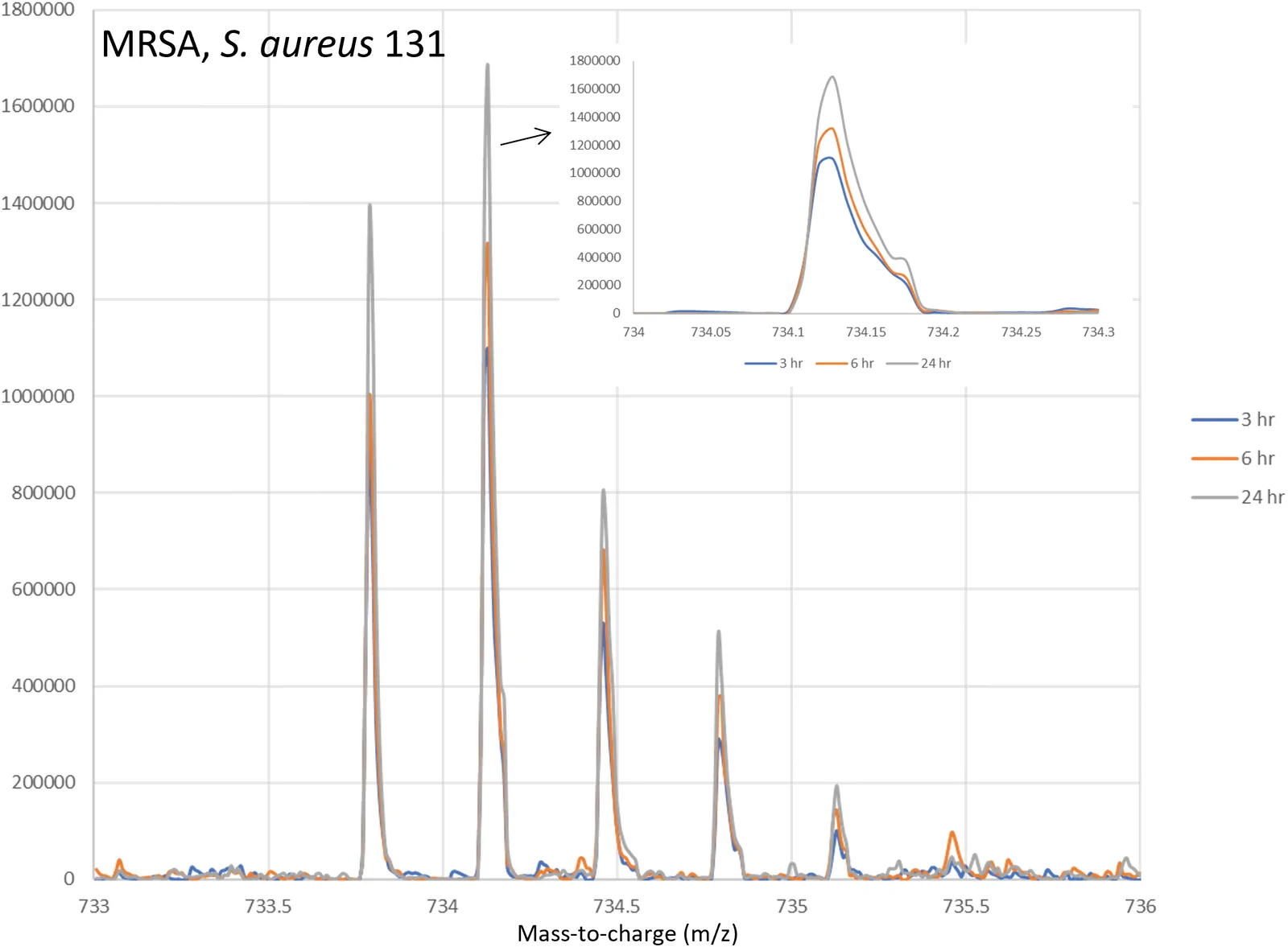
Comparison of the intensity at different incubation periods (3 h, 6 h, and 24 h) of FIA-MS of m/z 733.792. Inset is the zoomed-in region from 734-734.3 to provide an easier visualization of the increase over time.

### 3.3. Validation in Other Species and Clinical Isolates

The observed peaks (m/z 733.790 and m/z 691.613) were further validated by analyzing five different species from the same genus, including *S. caprae*, *S. epidermidis*, *S. lugdunensis*, *S. capitis*, and *S. warneri*, after incubating for 6 h. Neither of the m/z values were observed in any of these species, indicating specificity for MRSA and MSSA (**Figure 3**). For further validation of the study, a total of 20 clinical isolates (MSSA = 10, MRSA = 10) were also analyzed after incubating for 6 h. Data analysis revealed that the peak at m/z 691.613 (not shown) was found in all clinical isolates, while the peak at m/z 733.790 was only observed in MRSA clinical isolates and not in MSSA clinical isolates (**Supplementary Figure S2** and **S3**, respectively). The intensity of the peak at m/z 733.790 after incubation for 6 h was not very intense in the MRSA clinical isolates but was observed to be the peak of interest based on the m/z value, the charge state (+3), and its drift time (IM) (**Figure 4A–C**). The findings indicate that these peaks are only found in *S. aureus* and could be used to identify it as either MSSA or MRSA with m/z 691.613 and determine its resistance to methicillin (MRSA) using m/z 733.790. In the absence of chromatography or MS/MS, an additional dimension (i.e., IM) of analysis is important to reduce chemical noise and improve detection. In this case, the use of IM was key in reducing chemical noise, as can be observed when comparing the total ion signal as might be done in a typical FIA-HRMS experiment versus adding the IM dimension (**Figure 4B** and **C)**. When IM is added, then chemical noise is significantly reduced to show a clear isotopic pattern for m/z 733.790 (**Figure 4C**).

**Figure 3.**
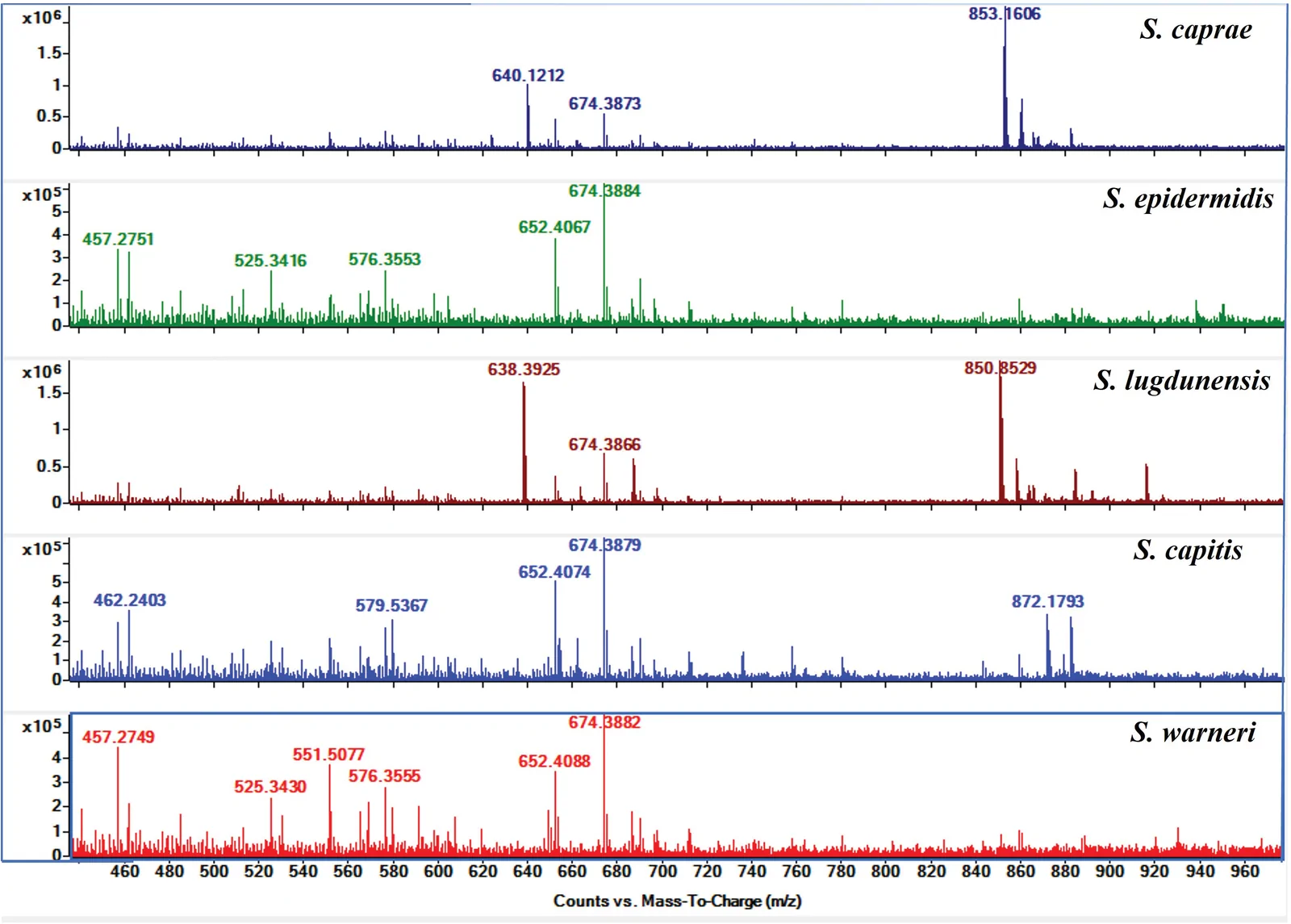
MS spectra of other species from the *Staphylococcus* genus after 6 h of incubation. No species showed the presence of the two peaks observed in MRSA.

**Figure 4.**
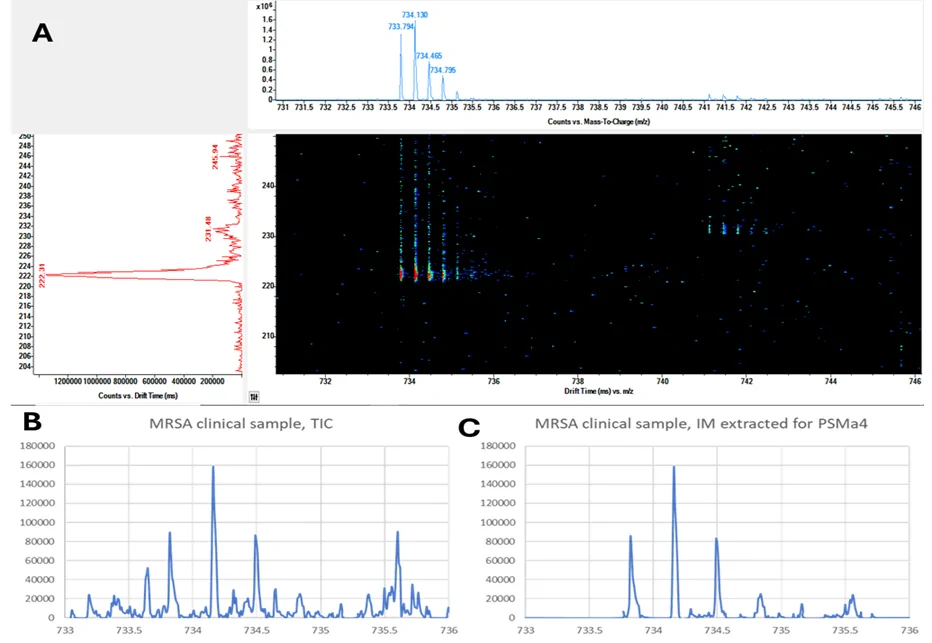
(**A**) Extracted mass spectrum and ion mobility spectra of the peak at m/z 733.792 with a drift time of 218–226 ms. (**B**) Comparison of the total ion counts for a clinical sample and (**C**) the same clinical sample extracted from the drift time of 218–226 with the mass range of 733–736. The reduction in chemical noise provided by ion mobility is evident and key to ensuring proper identification when LC or MS/MS is not used in connection with HRMS.

### 3.4. Initial Identification of Marker Peaks

Another important task in a biomarker study is to identify the proposed marker peaks. The peak at m/z 691.613 was not identified due to poor MS/MS fragmentation, while the ion at m/z 733.791 was identified using de novo sequencing by matching the fragments for the amino acid losses from the MS/MS spectrum. The approach led us to the identification of a known peptide, phenol-soluble modulin alpha 4 (PSMα4), with a sequence of 20 amino acids (MAIVGTIIKIIKAIIDIFAK) as shown (with different colors) in the annotated MS/MS spectrum (**Figure 5)**. The N-formylation is a common modification in bacterial proteins [28]. Hence, the sequence of the identified peptide has a monoisotopic mass of 2170.342 and is N-formylated, having a mass shift of 28 daltons (Da) to give a mass of 2198.347. Therefore, the [M + 3H]^3+^ peak of the peptide would appear at m/z 733.789 (theoretical), matching our observed m/z value. The remaining five amino acids are not presented in the spectrum due to the lack of sequence coverage. While the identified peptide has been frequently demonstrated for its role in *S. aureus* pathogenicity and virulence [29], we propose that the peak at m/z 733.792 could be used as a marker for rapid discrimination between MRSA and MSSA in clinical samples.

**Figure 5.**
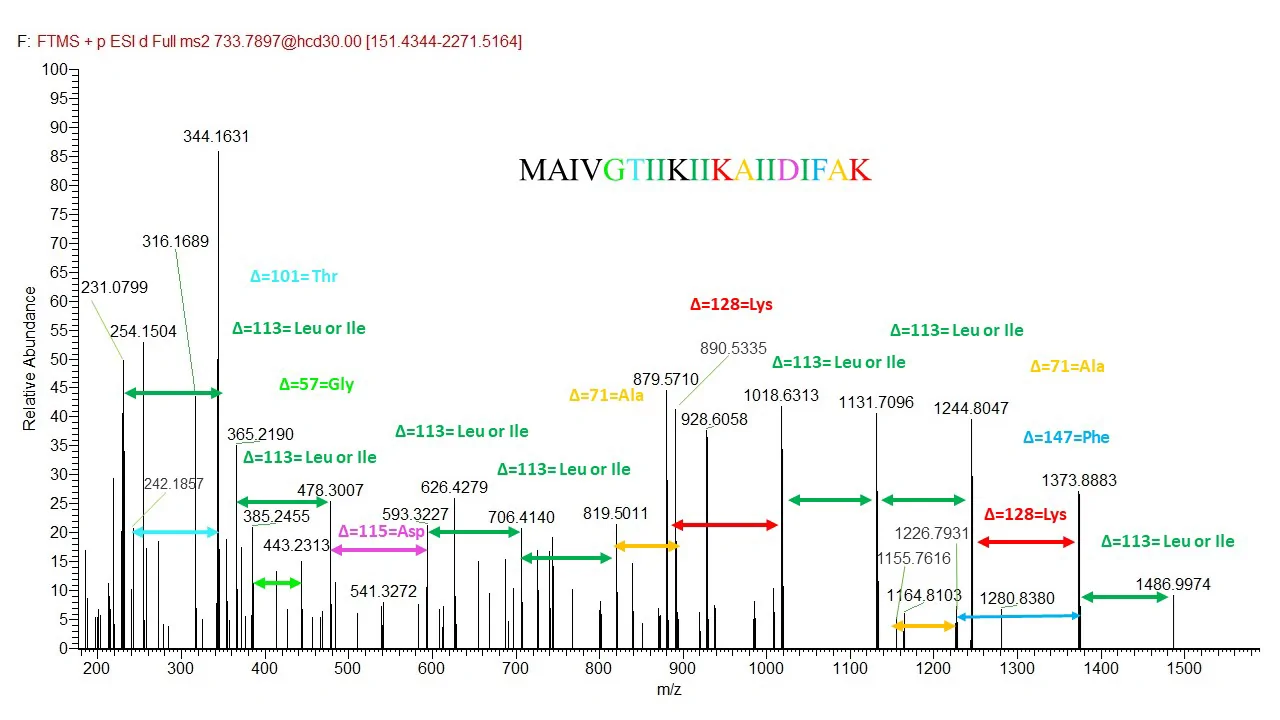
MS/MS fragmentation spectrum for the peak at m/z 733.790. Logical losses for amino acids are shown using the de novo sequencing method. The peak is identified as PSMα4. (The amino acids colored in black in the sequence were not present in the spectrum.)

### 3.5. Multivariate Statistical Analysis

For further validation of the proposed marker peak at m/z 733.790 in clinical isolates, multivariate statistical analysis was performed using MetaboAnalyst. The t-test analysis resulted in 34 significant features (*p* ≤ 0.05), which were further subjected to hierarchical clustering analysis for the comparison between the MSSA and MRSA strains, where only the top 30 are displayed in a heatmap (**Figure 6A**). The heatmap shows that the intensities of the features that correspond to PSMα4 (peaks at 733 and 734 from the isotopic pattern) are higher in MRSA strains compared with MSSA. We then conducted sPLS-DA to evaluate the variable selection of two-block datasets in a one-step procedure while integrating variables of two types, improving interpretability via graphical outputs. **Figure 6B** shows the sPLS-DA score plot that separates the two groups into two components based on the features, while **Figure 6C** shows the error rates of the sPLS-DA classification model. The model had a 5% error rate with two components, indicating strong performance. **Figure 6D** represents the VIP scores from the sPLS-DA and shows the variables that were either up- or downregulated. In addition to the peaks corresponding to PSMα4, the VIP score plot also shows a feature at m/z 769.1418, which we identified to be PSMα2 with a sequence of 21 amino acids (MGIIAGIIKFIKGLIEKFTGK) using the annotated MS/MS spectra in **Supplementary Figure S4**. The monoisotopic mass of this peptide from the sequence is 2276.369, while N-formylation causes a mass shift of 28 Da (2304.364). Therefore, the [M + 3H]^3+^ peak would give m/z 769.129 (theoretical), and our measured m/z was 769.142. This peptide has also been identified for its role in *S. aureus* pathogenicity and virulence [30]. The peaks at m/z 763.176 and 763.489 can be assigned to PSMα1 using [M + 3H]^3+^ (level 3 identification, the exact m/z of 763.121). We then returned to the incubation experiment to further validate the presence of PSMα1 and PSMα2 and show that both peaks are present at all time points but are observed at their highest at 6 h and 24 h (**Supplementary Figure S5**).

**Figure 6.**
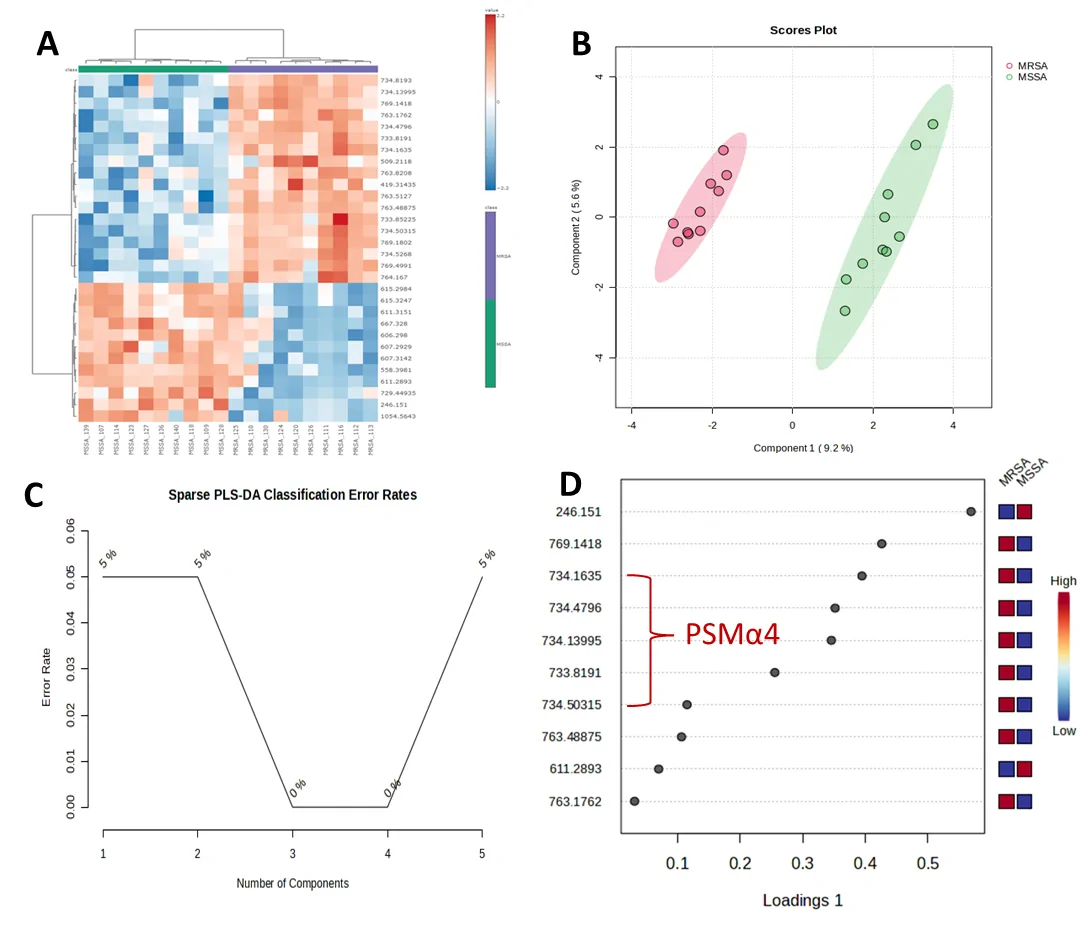
Multivariate statistical analysis of MSSA and MRSA clinical isolates. (**A**) Heatmap of the top 30 features from the t-test. (**B**) sPLS-DA score plot performed on the top features separated into two components. (**C**) Graph for sPLS-DA classification error rates. (**D**) VIP score plot from the sPLS-DA analysis.

The most significant VIP and the most significant from the t-test was m/z 246.151 (*p* = 2.78 × 10^⁻8^), and it was elevated in MSSA. A search for this m/z value in the Human Metabolome Database matched to S-3-oxodecanoyl cysteamine and several dipeptides (Asn-Leu, Ala-Arg, Val-Glu). The exact identification of this small molecule was not conducted to confirm the validity of the assignment. Our model was able to discriminate between both MRSA and MSSA based on several significant features observed from FIA-IM-MS. Hence, the statistical analysis also validated the efficacy of the initial proposed marker (m/z 733.790) for discriminating resistant strains from sensitive ones.

### 3.6. Comparison with Reported Methods

Several methods have been reported for the rapid discrimination of MRSA and MSSA strains of *S. aureus* using MS. A previously reported study showed the utilization of intact cell MS, in which a single bacterial colony was emulsified with a matrix, followed by MALDI-MS analysis, in which discrimination was based on the presence and absence of the number of peaks in specific mass ranges [31]. In another study, MALDI-TOF MS was utilized with support vector machine (SVM) analysis for the rapid discrimination of MSSA from MRSA. The study was also based on the presence of six peaks and their average intensities in both MRSA and MSSA samples [32]. Similarly, Kim et al. have reported the discriminatory model for 320 clinical (MRSA and MSSA) *S. aureus* isolates using PCR for staphylococcal cassette chromosome mec typing and MALDI-TOF MS for the identification of specific marker peaks. A total of 21 peaks were identified that were utilized for the discrimination [33]. In addition, Yu et al. reported MRSA discrimination using MALDI-TOF MS with a machine-learning approach [34]. More than 20,000 clinical isolates were used to build the model for the identification of MRSA and MSSA and their markers [34]. Another study also reported using MALDI-TOF MS–based machine learning for the early differentiation of MRSA from MSSA [35]. The use of a suitable matrix has been shown to enhance the identification of antibiotic-resistant bacteria—i.e., (E)-propyl α-cyano-4-hydroxyl cinnamylate (CHCA-C3) in this case [17]. All the reported studies utilizing MALDI-TOF MS for rapid discrimination between MRSA and MSSA are based on several unknown discriminatory peaks and machine-learning approaches that may make the analysis complex and ambiguous. Other non-MS methods, such as identification using flow cytometry (FCM) by the comparison of counts in paired broth cultures either containing or lacking oxacillin, have also been reported [36], as well as the use of multiplex loop-mediated isothermal amplification linked to a nanoparticle-based lateral flow biosensor (m-LAMP-LFB), surface-enhanced Raman spectroscopy (SERS), and deep-learning techniques [37, 38].

In comparison with the reported methods, our method is rapid, simple, and convenient and performs discrimination between MRSA and MSSA based on either a single peak (PSMα4) or several peaks associated with PSMs (PSMα1 and PSMα2). A detailed comparison of the current work with previously reported methods is presented in **Table 1**.

**Table 1.** Comparison of the present work with previously reported methods for their advantages and disadvantages.

Systems	Pros	Cons	Marker peaks (m/z)		Statistical tools	References
			MRSA	MSSA		
Assay 1: FIA-MS	-Both visual and statistical differentiation between spectra -Discrimination based on a single m/z -Discrimination can be achieved after a 6 h incubation -Identification of the marker peaks -Analysis of both standard strains and clinical isolates	-Limited number of clinical samples	733.790 763.170 769.142	——	Multivariate statistical analysis	Present work
Assay 2: intact cell-mass spectrometry	-Only examine intact bacterial surface chemistry -Visual discrimination	-Long incubation time -Discrimination based on several peaks	891, 1140, 1165, 1229, and 2127	2548 and 2647	——	[31]
Assay 3: MALDI-TOF MS	-Visual inspection of spectra -Protein identification	-Lengthy procedures -Require a prior conventional identification method	1888.1, 1935.9, 2760.3, 2867.9, 3044.2, and 4641.3 **Higher intensities**	**Lower intensities**	SVM	[32]
Assay 4: MALDI-TOF MS	-Large number of clinical isolates used	-Long incubation period -Use of PCR prior to biotyping -Use of deoxyribonucleic acid (DNA) extraction kits -Several peaks make it more ambiguous	2204, 2410, 2592, 4607, and 9216	2194, 2232, 2301, 2339, 2631, 2686, 3034, and 5509	SPSS, Pearson’s chi-square tests	[33]
Assay 5: MALDI-TOF MS	-Over 20,000 clinical MSSA and MRSA isolates used -machine learning algorithms	-LC-MS for protein identification -Tryptic digestion protocols	6,590 to 6,599 Da	——	Light gradient boosting machine	[34]
Assay 6: MALDI-TOF MS	-Use of synthesized matrix CHCA-C3 -Comparison between the spectra of MSSA and MRSA	-Several peaks make it more ambiguous -Spectral comparison	2306, 2322, 4821, and 9645	2306 and 2322	Principal component analysis	[17]
Assay 7: FCM	-Lower incubation periods -Large number of isolates	-Utilize side-scatter and fluorescence intensity differences using FCM	——	——	SigmaPlot, means, and confidence intervals	[36]
Assay 8: m-LAMP-LFB	-Detect MRSA-specific primers based on the *femA* and *mecA* genes	-Primer synthesis -Genomic DNA template preparation	——	——	——	[37]
Surface enhanced Raman spectroscopy (SERS)	-Deep-learning algorithms -Differences between the band intensities of MRSA and MSSA	-AgNPs synthesis -High signal-to-noise ratio	——	——	Mann-Whitney U test	[38]

## 4. Discussion

In this study, we developed a rapid FIA-IM-HRMS method for discrimination between MSSA and MRSA strains after incubating for 6 h and proposed a marker peak at m/z 733.790, which can be utilized for this discrimination. We also presented work from multivariate statistical analysis that identified two other peaks, which were discriminatory for MRSA versus MSSA at m/z 763.176 and 769.180, independently of the peak at 733.790. The marker peaks were identified as peptides from a peptide family known as PSMs. Previously, a similar approach was used for typing and discrimination between MSSA and MRSA based on some characteristic peaks using intact cell MS. However, no peaks were identified for any specific compounds [31]. Recently, an untargeted lipidomic study has also been conducted to track differences between MSSA and MRSA strains, in which three lipid species were observed to differ between the two by using UHPLC-MS [39].

*S. aureus* can produce a variety of pathogenic elements that harm its host and circumvent immunity, accounting for its ability to survive in the human host [40]. Numerous pathogenic and virulent agents, including enterotoxins, α-toxins, toxic shock syndrome toxins, and PSMs, can be produced by *S. aureus* [41]. PSMs, including δ-toxin, can make up 60% of all proteins produced in a culture grown overnight and constitute the most prominent peptides. The family of PSM peptides plays an important role in the pathogenicity of *S. aureus* [42, 43].

The formation of biofilm and virulence in *S. aureus* is regulated by the family of these small toxin peptides known as PSMs [29, 44]. The PSMs contribute to pathogenesis in a variety of ways, including killing red and white blood cells, regulating both adaptive and innate immune responses, promoting skin colonization, and contributing to the emergence of infections associated with biofilms [29].

The peptide identified as PSMα4 in this study was previously reported by Kocurek et al. in the *S. aureus* strain (MSSA 476) as a (+3) charged peak at m/z 733.7894 and has been shown to cause the lysis of human neutrophils [45]. Its increased production is reported to be associated with high virulence in specific community-associated MRSA strains [42]. The peptide PSMα4 from the supernatant of the USA300 strain culture has been shown to induce the release of heparin-binding protein (HBP) from polymorphonuclear neutrophils (PMNs), and this HBP release from PMNs induced by PSMα4 causes the vascular leakage [46].

Despite the known association of PSMα4 with pathogenicity, there is a lack of reports on its association with *S. aureus* antibiotic resistance. Future research should evaluate the effectiveness of our method employing direct inoculation and broth extraction, which could improve workflow efficiency and enable integration with automated systems. Such advancements would improve utility compared to chromogenic agar by combining rapid detection with higher throughput and reduced labor. In addition to genetic assays, protein-based detection of the *mecA* gene product PBP2a has been explored as a diagnostic marker for MRSA, using immunochromatographic and latex agglutination assays [47, 48]. While our method focuses on small molecules and peptide signatures, these approaches highlight complementary strategies that may further support rapid MRSA detection and reveal the association of the proposed marker peptide with the development of AMR among *S. aureus* strains.

## 5. Conclusion

Our study revealed a rapid assay for discrimination between MRSA and MSSA after 6 h of incubation. The developed method using FIA-IM-HRMS has substantially improved the detection time for discrimination between resistant and sensitive strains of *S. aureus*, which may provide an opportunity for early intervention and guided therapy. The IM dimension was key in reducing chemical noise to enable detection in the absence of chromatography. The proposed biomarker peaks that could be used for discrimination have also been identified from the family of PSMs, which may play a pivotal role in the early identification and detection of AMR, and future work on developing a quantitative assay could help to define clinical reference ranges needed for diagnostics. The role of the identified peptides in the development of AMR is not fully understood. However, future work on its role and association in the development of AMR in clinical isolates could be a potential breakthrough in early and rapid AMR diagnostics.

## Data Availability

The data that support the findings of this study are available from the corresponding author upon reasonable request.

## Abbreviations

AMR: Antimicrobial resistance
ATCC: American Type Culture Collection
CHCA-C3: (E)-propyl α-cyano-4-hydroxyl cinnamylate
Da: Dalton
DNA: Deoxyribonucleic acid
ESI: Electrospray ionization
FA: Formic acid
FCM: Flow cytometry
FIA: Flow injection analysis
FIA-IM-HRMS: Flow injection analysis–ion mobility–high-resolution mass spectrometry
FIA-IM-MS: Flow injection analysis–ion mobility–mass spectrometry
FIA-MS: Flow injection analysis mass spectrometry
HBP: Heparin-binding protein
HPLC: High-performance liquid chromatography
HR-IM: High-resolution ion mobility
HRMS: High-resolution mass spectrometry
H_2_O: Water
IM: Ion mobility
IMS: Ion mobility spectrometry
LC: Liquid chromatography
MALDI-TOF MS: Matrix-assisted laser desorption ionization time-of-flight mass spectrometry
m-LAMP-LFB: Multiplex loop-mediated isothermal amplification linked to a nanoparticle-based lateral flow biosensor
MRSA: Methicillin-resistant *Staphylococcus aureus*
MS: Mass spectrometry
MS/MS: Tandem mass spectrometry
MSSA: Methicillin-susceptible *Staphylococcus aureus*
PCR: Polymerase chain reaction
PMNs: Polymorphonuclear neutrophils
PSMs: Phenol-soluble modulins
QTOF: Quadrupole time-of-flight
RF: Radio frequency
SERS: Surface-enhanced Raman spectroscopy
SLIM: Structures for lossless ion manipulation
sPLS-DA: Sparse partial least squares discriminant analysis
SVM: Support vector machine
TIC: Total ion counts
TOF: Time-of-flight
TSB: Tryptic soy broth
TWIMS: Traveling wave ion mobility spectrometry
UHPLC: Ultrahigh-performance liquid chromatography
VIP: Variable importance in projection

## Supplementary Material

Supplemental Material Available at https://doi.org/10.21627/vx9rfz41
